# A Novel Strategy for the Detection of Semicarbazide in Crustaceans by Modified QuEChERS Coupled with Hydrophilic Interaction Liquid Chromatography–Tandem Mass Spectrometry

**DOI:** 10.3390/foods14030541

**Published:** 2025-02-06

**Authors:** Siyuan Wu, Yang Feng, Shengjun Chen, Yongqiang Zhao, Chunsheng Li, Jianchao Deng, Di Wang

**Affiliations:** 1 College of Food Science, Shanghai Ocean University, Shanghai 201306, China; 15737010880@163.com; 2 Key Laboratory of Aquatic Product, Ministry of Agriculture and Rural Affairs, National R&D Center for Aquatic Product Processing, South China Sea Fisheries Research Institute, Chinese Academy of Fishery Sciences, Guangzhou 510330, China; zhaoyq@scsfri.ac.cn (Y.Z.); lichunsheng@scsfri.ac.cn (C.L.); dengjianchao@scsfri.ac.cn (J.D.); wangdi@scsfri.ac.cn (D.W.); 3 Key Laboratory of Efficient Utilization and Processing of Marine Fishery Resources of Hainan Province, Sanya Tropical Fisheries Research Institute, Sanya 572426, China

**Keywords:** semicarbazide, crustaceans, shrimp, QuEChERS, HILIC-MS/MS, *Macrobrachium rosenbergii*, nitrofurazone, kinetic model

## Abstract

Semicarbazide (SEM), a metabolite of nitrofurazone (NFZ), is widely used to detect the illegal application of NFZ in crustaceans. The conventional detection method involves chemical derivatization combined with reversed-phase liquid chromatography–tandem mass spectrometry (RPLC-MS/MS), which is both complex and time-consuming. To address this limitation, a more efficient approach was developed for SEM detection. This study introduces a modified QuEChERS pretreatment method coupled with hydrophilic interaction liquid chromatography–tandem mass spectrometry (HILIC-MS/MS) for detecting SEM in crustaceans. The proposed method is simple, fast, and highly accurate, making it universally applicable for SEM detection in crustaceans. Additionally, the method was applied to investigate NFZ metabolism in *Macrobrachium rosenbergii* with a kinetic model. The findings suggested a plausible mechanism for the absorption of NFZ and its subsequent transfer from meat to the shell. In conclusion, this study provides a simple and rapid technique for SEM detection in crustaceans with immense application value.

## 1. Introduction

Semicarbazide (SEM), an aminoguanidine compound, is known for its moderate accumulation toxicity, particularly its reproductive toxicity and mutagenic effects [1]. Sources of SEM in foodstuffs originate from the following: (i) exogenous chemical conversions (e.g., thermal degradation of azodicarbonamide in flour products), (ii) environmental contaminants (e.g., hypochlorite disinfection byproducts), and (iii) bioaccumulation from aquaculture (e.g., arginine and urea-related metabolism in crustaceans) [2,3,4]. Additionally, SEM has been detected in honey and the plastic seals of certain food containers [5,6]. While the aforementioned SEM levels in natural foods are relatively low, elevated levels are often linked to nitrofurazone (NFZ) metabolism.

NFZ, a cost-effective, broad-spectrum, redox-active antimicrobial agent, effectively treats bacterial diseases in livestock and aquatic animals [7]. However, due to the toxicity of its secondary metabolites, such as SEM, the Food and Drug Administration (FDA) and the European Union banned NFZ as an antimicrobial treatment in aquaculture in 1955 [8]. Similarly, the Ministry of Agriculture and Rural Affairs of China, in announcement No. 250, classified NFZ as a prohibited substance for food animals.

To detect NFZ usage in food sources, researchers have proposed several target compounds alongside the internationally recognized SEM standard. Techniques have been developed to identify NFZ secondary metabolites, such as 5-nitro-2-furaldehyde, but the rapid metabolism of the parent drug in organisms limits the practicality of these methods. SEM can bind to proteins, forming stable polymers. Consequently, SEM is widely used as a characteristic secondary metabolite of NFZ, with detection limits established by regulatory agencies in China, the United States, and the European Union.

Due to its amide and urea bonds, SEM exhibits appreciable responses in mass spectrometry (MS) analysis. However, its high polarity causes it to elute during the dead time of reversed-phase liquid chromatography–tandem mass spectrometry (RPLC-MS/MS), resulting in strong matrix effects that suppress detection signals. This necessitates derivatization for SEM detection. Currently, most analytical methods for SEM are similar to those of Verdon et al. and involve three stages: sample pretreatment, derivatization, and detection. During sample pretreatment, acidic hydrolysis, often using acidified acetonitrile, can facilitate SEM release from proteins. Derivatization typically requires neutralizing the extraction solution’s pH in order to enhance SEM’s hydrophobicity, followed by using 2-nitrobenzaldehyde (NBA) for overnight derivatization. Liquid–liquid extraction (LLE) is then used to collect the derivatized products, with additional operations for fat removal. Data analysis is conducted using RPLC-MS/MS in multiple reaction monitoring (MRM) mode [9]. Although researchers have optimized various aspects, such as pretreatment, derivatization time, and mobile phase composition, these processes remain complex and time-consuming [10].

The Quick, Easy, Cheap, Effective, Rugged, and Safe (QuEChERS) method enables rapid, high-throughput sample extraction and aligns with green chemistry principles. This method is ideal for analyzing multi-residue pesticides in various food matrices, offering efficiency, reduced costs, and minimal environmental impact [11]. Hydrophilic interaction liquid chromatography (HILIC), a type of normal-phase chromatography, effectively separates polar compounds [12]. Leveraging these characteristics, this study developed a modified QuEChERS pretreatment method coupled with hydrophilic interaction liquid chromatography–tandem mass spectrometry (HILIC-MS/MS) to detect SEM in crustaceans. This method offers universal applicability for SEM detection in crustaceans. Additionally, it was employed to investigate SEM metabolism in *Macrobrachium rosenbergii* under NFZ-treated farming conditions. This approach is expected to provide valuable methodological support for SEM content analysis and mechanism research in crustaceans.

## 2. Materials and Methods

### 2.1. Chemicals and Reagents

Semicarbazide standard (purity > 99.8%) was obtained from Macklin (Shanghai, China). Nitrofurazone (NFZ, purity > 99.5%) and isotopically labeled semicarbazide hydrochloride (^13^C-^15^N_2_-SEM·HCl) were purchased from Anpu (Shanghai, China). Mass spectrometry-grade formic acid (HCOOH) was acquired from Thermo Scientific (Waltham, MA, USA). Sodium sulfate (Na_2_SO_4_), magnesium sulfate (MgSO_4_), ammonium acetate (NH_4_OAc), and sodium chloride (NaCl) were obtained from Sinopharm Chemical Reagent Co., Ltd. (Shanghai, China). LC-MS-grade acetonitrile (ACN) and C18 solid-phase extraction columns were purchased from Waters Corporation (Milford, MA, USA). Primary secondary amine (PSA) solid-phase extraction columns were sourced from Sinopharm (Shanghai, China). Enhanced matrix removal (EMR)-lipid purification tubes were obtained from Agilent Technologies (Santa Clara, CA, USA). Ultrapure water was prepared using a Milli-Q-Plus ultrapure water system (Millipore, Bedford, MA, USA).

### 2.2. Stock Solution Preparation

A total of 100 mg of the SEM standard was dissolved in 100 mL of acetonitrile–water (1:1, *v/v*) to prepare a stock solution (1 mg/mL). This stock solution was subsequently diluted to 10 μg/mL using acetonitrile to create the working solution. All low-concentration solutions are obtained by diluting the working solution with acetonitrile. Similarly, the SEM internal standard stock solution was prepared by dissolving the isotopically labeled semicarbazide hydrochloride in acetonitrile at a concentration of 1 mg/mL and then diluting the solution to 10 μg/mL using acetonitrile to create the working solution. The stock solution was stored at −20 °C to avoid potential hydrolysis, while the working solution was freshly prepared for each use.

### 2.3. QuEChERS Pretreatment of Crustacean Samples

The shrimp samples consisted of the abdominal muscle (shrimp meat) and abdominal shell (shrimp shell), which were processed using a homogenizer (10,000 rpm for 2 min). For shrimp meat, 3 g of the sample was accurately weighed and placed in a 50 mL polypropylene centrifuge tube. To this, 15 mL of acidified acetonitrile (1% formic acid) and 100 μL of internal standard solution (10 μg/mL) were added. After vortexing for 30 s, 4 g of ammonium acetate was introduced, followed by vortex mixing at 2500 rpm for 30 s and centrifugation (4000 rpm, 10 min). The supernatant was collected and transported into a dispersive solid-phase extraction (dSPE) purification tube containing 2 g of material. After vortexing for another 30 s, low-speed centrifugation (1000 rpm, 5 min) was performed to collect the supernatant. The supernatant was dried under nitrogen in a water bath at 40 °C, re-dissolved in 1 mL of acidified acetonitrile, filtered through a 0.22 μm membrane, and analyzed using LC-MS/MS with hydrophilic interaction performance with the overall methodology referred to as HILIC-MS/MS. For shrimp shell analysis, 2 g of shrimp shell was accurately weighed, cut into small pieces, and placed in a 50 mL polypropylene centrifuge tube. To this, 20 mL of acidified acetonitrile (1% formic acid) and 100 μL of internal standard solution (10 μg/mL) were added. The following steps are identical to the shrimp meat method. The crab meat processing was similar to the shrimp meat method, with the key difference being the exclusion of crab roe during collection.

### 2.4. Equipment and Chromatographic Conditions for HILIC-MS/MS Analysis

The Waters Acquity UPLC^®^ BEH HILIC column (1.8 μm, 100 mm × 2.1 mm) was used for compound separation, with the temperature maintained at 40 °C. The mobile phases were water (1% formic acid; phase A) and acetonitrile (0.1% formic acid; phase B). The elution gradient was as follows: 0–3 min, 100% B; 3–4.5 min, 100% to 80% B; 4.5–5.5 min, 80% to 60% B; 5.5–8.5 min, 60% B; 8–9 min, 60% to 100% B; and 10–15 min, 100% B. The flow rate was set at 0.5 mL/min. Quantification was performed using the ion with the highest abundance under positive ion conditions (SEM, 76.1 > 30.8; ^13^C-^15^N_2_-SEM, 79.0 > 33.0). Qualitative analysis was conducted using characteristic ion fragments (SEM, 76.1 > 58.8; ^13^C-^15^N_2_-SEM, 79.0 > 61.8). The capillary voltage was set to 0.8 kV. The cone and collision voltages were both set at 12 V. The ion source temperature was maintained at 300 °C, with a desolvation gas flow of 1000 L/h (N_2_) and a cone gas flow of 20 L/h (N_2_). The automatic sampler temperature was set to room temperature, and the injection volume was 0.5 μL.

### 2.5. Method Validation

The mixed standard working solutions of SEM and ^13^C-^15^N_2_-SEM were prepared with concentration ratios of 0.01, 0.02, 0.05, 0.1, 0.2, 0.5, 1.0, 2.0, 5.0, 10.0, 20.0, and 50.0 (with the concentration of ^13^C-^15^N_2_-SEM fixed at 1 μg/mL). The standard curve was plotted with the concentration ratio on the *x*-axis and the response ratio on the *y*-axis. The concentration corresponding to a signal/base ratio of 3 was defined as the limit of detection (LOD), while a signal/base ratio of 10 was used to define the limit of quantification (LOQ). The sensitivity was represented by the slope of the calibration curve.

The extraction recovery, matrix effect, and recovery were evaluated by a previously reported method with some modifications [13]. The SEM concentration measured when shrimp samples were replaced with pure solvent and was defined as C_spiked_, the SEM concentration detected in unspiked shrimp samples was defined as C_endogenous_, the SEM concentration detected in shrimp samples with standards added before pretreatment was defined as C_pre-spike_, and the SEM concentration detected in extracts obtained after pretreatment with standards added was defined as C_post-spiked_. The formulas for calculating the recovery and matrix effect are as follows:

Extraction efficiency (%) = [(C_pre-spike_ − C_endogenous_)/(C_post-spike_ − C_endogenous_)] × 100

Matrix effect (%) = [(C_post-spike_ − C_endogenous_)/(C_spiked_)] × 100

Recovery (%) = [(C_pre-spike_ − C_endogenous_)/(C_spiked_)] × 100

Precision was expressed as intra-day and inter-day precision. The mixed standard solutions of SEM and ^13^C-^15^N_2_-SEM were used with concentration ratios of 0.1, 1.0, and 10, and each sample was analyzed in triplicate. Injections were performed three times at different intervals on the same day and three times on different days within the same week, with the coefficient of variation (CV) examined.

### 2.6. Detection of SEM in Crustaceans

Six crustacean species (each independent organism contains three) were obtained from a local seafood supplier in the Haizhu District of Guangzhou City, China. Samples were stored at −80 °C for subsequent analysis (detailed information about the crustacean samples is listed in the Supplementary Materials). SEM detection was performed following the aforementioned method. All animal experiments were approved by the Ethics Committee of the South China Sea Institute of Aquatic Research, Chinese Academy of Fishery Sciences, in compliance with ethical guidelines and regulations.

### 2.7. Application to NFZ-Treated Macrobrachium rosenbergii Samples

The immersion method was used to assess the impact of a short-term NFZ bath on SEM production in *Macrobrachium rosenbergii* (detailed descriptions of the sample information and NFZ-treatment procedure are listed in the Supplementary Materials). Before the experiment, shrimp fasted for two days were evenly divided into two groups, each containing 21 shrimp. The control group received no NFZ, while the experimental group was immersed in an NFZ solution (10 mg/L) for 2 h. Following treatment, the shrimp were transferred to clean water for normal cultivation. Samples were collected at 0, 4, 12, 36, 108, 324, and 972 h, with three shrimp sampled at each time point.

The collected shrimp were rapidly frozen using liquid nitrogen and stored at −80 °C. Prior to analysis, shrimp were slightly thawed to separate meat from shells. The abdominal muscle served as the sampling portion for shrimp meat, while the abdominal exoskeleton was used for shrimp shells. Samples were processed using a homogenizer and accurately weighed for detection and analysis.

### 2.8. Kinetics Study for SEM

The oral two-compartmental analysis was performed to calculate pharmacokinetic parameters using Drug and Statistics 2.0 (DAS 2.0) software (Mathematical Pharmacology Professional Committee of China) [14]. In this model, the drug enters the central compartment from the absorption site via the absorption rate constant (Ka), transfers to the peripheral compartment through K12, exchanges between the two compartments via K21, and is metabolized and eliminated from the central compartment through the elimination constant (K10). The model equation is as follows:

$$c\left(t\right)=Ae^{-\alpha t}+Be^{-\beta t}-Ke^{-k_{a}t}$$

where

- A: initial concentration or total amount of the drug in the central compartment;
- *α*: transfer rate constant from the central compartment to the peripheral compartment;
- B: initial concentration or total amount of the drug in the peripheral compartment;
- *β*: transfer rate constant from the peripheral compartment to the central compartment;
- K: elimination rate constant of the drug in the central compartment;
- ka: absorption rate constant of the drug.

### 2.9. Statistical Analysis

The experimental data were processed with SPSS 19.0 software (SPSS Inc., Chicago, IL, USA). We used a paired *t*-test to evaluate the difference between samples. All *p* values were two-sided, and *p* values < 0.05 were considered to have statistical significance.

## 3. Results and Discussion

### 3.1. Optimization of QuEChERS Pretreatment

**Figure 1:**
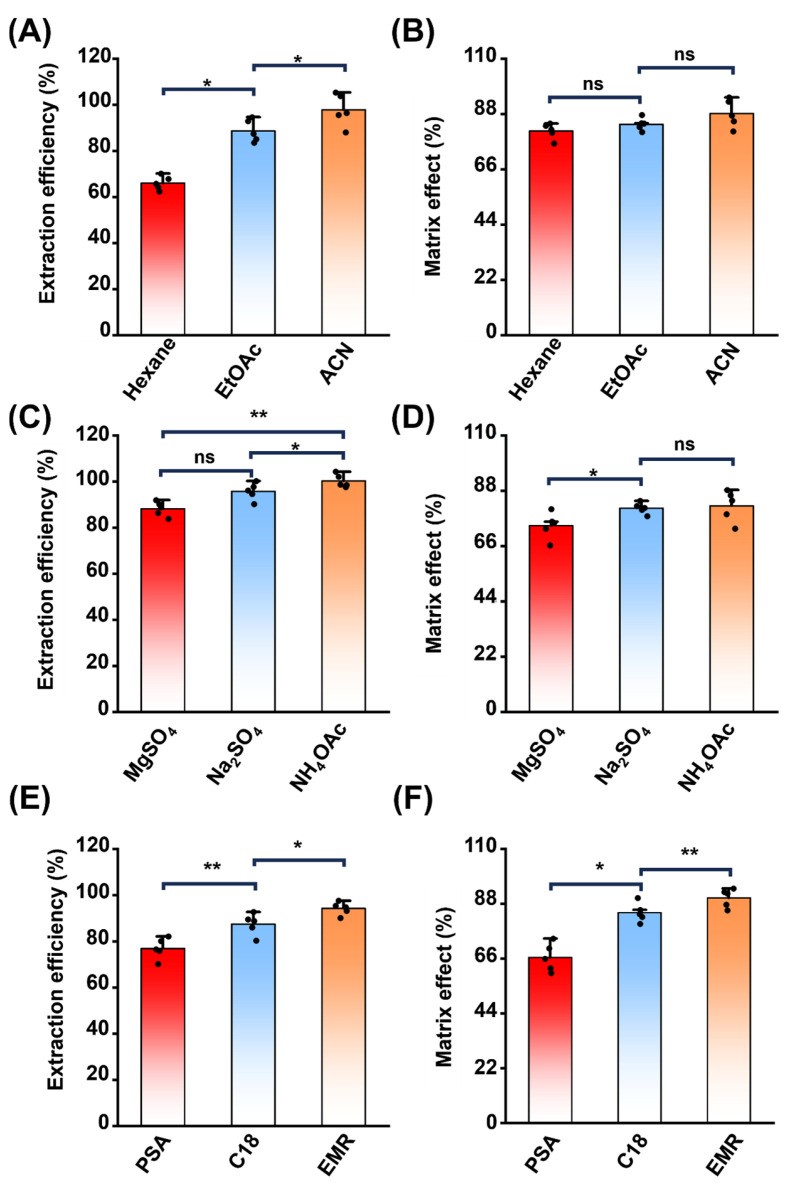
Optimization of QuEChERS pretreatment. (**A**,**B**) Extraction efficiency (**A**) and matrix effect (**B**) of hexane, acetonitrile (ACN), and ethyl acetate (EtOAc) as extraction solvents. (**C**,**D**) Extraction efficiency (**C**) and matrix effect (**D**) of MgSO_4_, Na_2_SO_4_, and NH_4_OAc as dehydrating reagents. (**E**,**F**) Extraction efficiency (**E**) and matrix effect (**F**) of PSA, C18, and EMR as types of dSPE. Asterisks denote statistical significance levels: * *p* < 0.05, ** *p* < 0.01, and ns = not significant (*p* ≥ 0.05).

SEM, a secondary metabolite of NFZ, can bind to proteins or peptides and form Schiff base adducts with carbohydrates, aldehydes, and ketones in the matrix. Mild acidification of samples before analysis enhances the release of SEM from the matrix [15]. Therefore, this study utilized the liquid extraction method to compare the extraction efficiencies of 1% formic acid-acidified n-hexane, ethyl acetate, and acetonitrile (Figure 1A,B). Hexane minimizes the influence of polysaccharides on the extraction efficiency and has proven effective for SEM extraction from substances such as flour and honey [16]. Acetonitrile and ethyl acetate, with higher polarity than n-hexane, more effectively dissolve polar substances, mitigating the effects of high fat and protein content in samples. This explains the superior extraction efficiency of acetonitrile and ethyl acetate compared to n-hexane. Additionally, acetonitrile, being completely miscible with water, enhances the extraction of water-soluble compounds and is commonly used in QuEChERS for pesticide residue analysis. Its effectiveness in precipitating proteins in aquatic products improves recovery and precision [17]. Subsequent analysis of the matrix effect showed no statistically significant differences among the three solvents. Thus, acetonitrile was chosen as the extraction solvent for shrimp samples to improve extraction selectivity. During shrimp shell processing, it was observed that the shells are more difficult to homogenize than shrimp meat, and their surface area in contact with the extraction solution is relatively smaller. This suggested that a larger volume of acetonitrile is necessary to achieve a satisfactory recovery for bound SEM. The recovery increased with increasing acetonitrile volume before stabilizing. Based on these results, the optimal extraction volume for shrimp shells was determined to be 20 mL (Figure S1).

After selecting the extraction solvent, various dehydrating agents were compared. In the QuEChERS method, inorganic salts such as MgSO_4_, Na_2_SO_4_, and NH_4_OAc are commonly used to facilitate phase separation and protein precipitation, enhancing extraction efficiency [18]. This research evaluated the impact of these reagents, combined with NaCl, on phase separation and extraction, focusing on recovery and the matrix effect. Fixed standard concentrations were added to the acetonitrile extract of shrimp samples, followed by a methodological comparison of dehydrating agents. NH_4_OAC demonstrated a superior extraction efficiency and matrix effect compared to MgSO_4_ and Na_2_SO_4_ (Figure 1C,D), likely due to the unique endothermic dissolution mechanism of ammonium salts. Furthermore, MgSO_4_ and Na_2_SO_4_, as low vapor pressure salts, often deposit on the MS ion source surface and accumulate inside the analyzer, reducing instrument performance. Therefore, volatile NH_4_OAc was selected as the dehydrating agent for this study.

The efficacy of dSPE in the QuEChERS method was further evaluated, focusing on the extraction performance of different sorbents, including PSA, C18, and EMR (Figure 1E,F). The type of filler used in dSPE significantly influenced the extraction efficiency and matrix effect [19]. EMR demonstrated the highest extraction efficiency and matrix effect, followed by PSA and C18. The PSA fillers, containing primary and secondary amines, exhibit weak anion exchange capabilities. These fillers effectively remove interfering substances, such as fatty acids, organic acids, polar pigments, and sugars, while mitigating the matrix effect [11]. Conversely, C18 fillers, classified as reverse-phase fillers, rely on absorption rather than electro-affinity for enriching target substances [20]. This approach poses challenges for the small SEM molecule, leading to a lower extraction efficiency and greater variability in the data due to the matrix effect. The QuEChERS dSPE EMR-lipid purification tube contains a variety of mixed purification agents that utilize size exclusion, as well as hydrophilic and hydrophobic interactions. This allows for the effective simultaneous removal of interfering substances with diverse properties [21,22]. In addition, EMR selectively binds to lipid and polar substances through hydrophobic interactions and hydrogen bonding, facilitating their enrichment and subsequent removal during centrifugation [23]. In contrast, most chemical contaminants structurally distinct from lipids do not bind to EMR-lipid dSPE [12,24]. It is worth noting that the differences in extraction efficiency and the matrix effect between PSA and EMR were less pronounced than those observed when compared to C18. Based on the mechanism of the three methods, it was speculated that the variations in the extraction efficiency and matrix effect were influenced by lipid content and polar substances [25].

### 3.2. Optimization of HILIC-MS/MS Analytical Conditions

**Figure 2:**
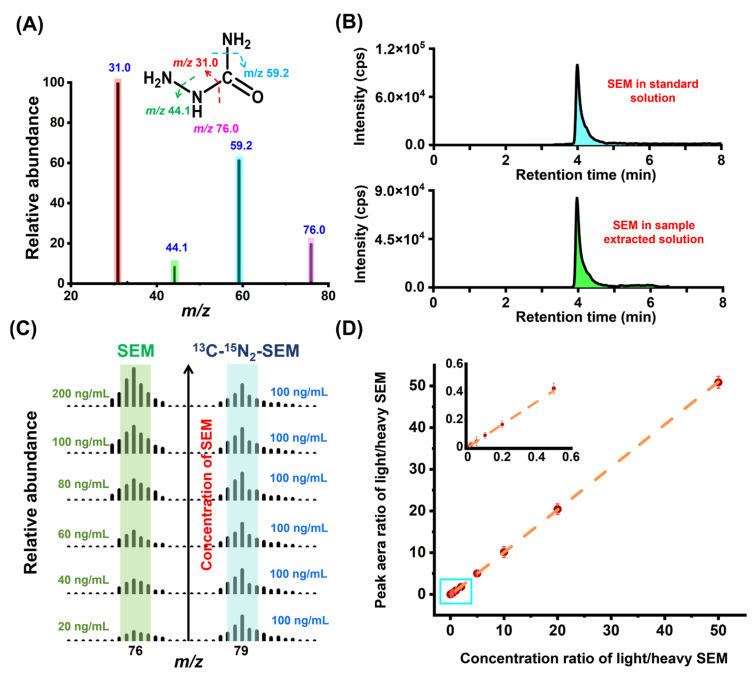
Establishment of QuEChERS coupled with HILIC-MS/MS method for SEM analysis. (**A**) Chemical structure and mass spectrum of SEM. (**B**) Extracted ion chromatograms of SEM in standard and sample extraction solutions. (**C**) Mass spectra of SEM and ^13^C-^15^N_2_-SEM at different concentrations. (**D**) Internal calibration curves for quantification of SEM.

The MS parameters for SEM and ^13^C-^15^N_2_-SEM were systematically optimized in this study. A standard solution with a concentration of 100 ng/mL was used, and the cone voltage and collision voltage were fine-tuned in combination mode. The ion fragments with the maximal responses were designated as quantitative ions. Lower-responding fragment ions were used as qualitative ions. The structural representation of SEM and its fragmentation behavior are shown in Figure 2A. The optimized MS parameters, including the parent ions, daughter ions, cone voltage, and collision voltage for SEM and ^13^C-^15^N_2_-SEM, are detailed in Table S1. The hydrazine group in the SEM structure contributes to its strong hydrophilicity, resulting in weak retention on reverse-phase chromatography columns. A preliminary test using the Waters Acquity UPLC^®^ C18 column (1.8 μm, 150 mm × 2.1 mm) confirmed that SEM elutes near the dead time (Figure S2). Significant changes in response values were observed in actual samples, likely due to polar co-eluting matrix components. Similarly, Stadler et al. reported challenges in SEM analysis during LC-MS/MS studies due to matrix co-extracts, leading to retention time shifts and false positives [5]. They emphasized that effective chromatographic separation is essential for accurate identification of SEM.

The HILIC column was selected to separate polar matrix components in the sample extract, optimizing the chromatographic behavior and response. During method optimization, the peak shape was evaluated using 1% formic acid or 10 mM ammonium acetate as the aqueous phase. Ultimately, 1% formic acid was chosen due to its superior peak shape. To stabilize the mobile phase pH, 0.1% formic acid-acidified acetonitrile was employed, effectively minimizing solvent effects and improving the chromatographic peak shape. Under optimized conditions, SEM exhibited strong retention in the HILIC column. To facilitate proper elution, the flow rate was increased from 0.3 mL/min to 0.5 mL/min, and the injection volume was reduced to 0.5 μL. Additionally, to mitigate the matrix effect on MS measurements before and after SEM peaks, the flow path was diverted to “Waste” at −0.5 min and +0.5 min around SEM’s retention time. The extracted ion chromatograms of SEM in the standard and matrix extraction solutions after optimization are presented in Figure 2B.

### 3.3. Validation of QuEChERS-HILIC-MS/MS Method

The feasibility of the proposed QuEChERS-HILIC-MS/MS method was assessed by evaluating the extraction efficiency, matrix effect, recovery, and precision with the conventional method. Standard solutions of SEM at 1 ng/mL, 10 ng/mL, and 100 ng/mL (low, medium, and high concentrations, respectively) were prepared and added to the samples for detection (n = 5). The QuEChERS-HILIC-MS/MS method exhibited extraction efficiencies of 78.2% to 95.4% across the three SEM concentration levels, with the matrix effect resulting in response losses of 7.9% to 24.3%. Purifying high concentrations of hydrophilic matrices from co-extraction without losing the analytes posed challenges. These findings aligned with the matrix complexity observed in shrimp samples. Despite this, the overall recovery and precision of the method met established methodological requirements. The recovery for the conventional method and our established method were 78.8% to 92.7% and 76.2% to 96.7%, respectively. Both methods demonstrated good precision at all three concentration levels, with coefficients of variation below 10%, meeting methodological standards (Table S2).

To further validate the method, varying SEM concentrations were added alongside a fixed concentration of ^13^C-^15^N_2_-SEM (100 ng/mL) to a blank matrix. These solutions were mixed in equal volumes and analyzed to compare the peak areas of the two analytes (Figure 2C). The results indicate that the response intensity of the two analytes with different *m/z* ratios is proportional to their concentrations. Using the same methodology, mixed solutions of SEM and ^13^C-^15^N_2_-SEM (100 ng/mL) with concentration ratios of 0.01, 0.02, 0.05, 0.1, 0.2, 0.5, 1.0, 2.0, 5.0, 10.0, 20.0, and 50.0 were prepared and subsequently introduced into a blank matrix. A standard curve was plotted with concentration ratios on the *x*-axis and peak area ratios on the *y*-axis. The method demonstrated excellent linearity, with calibration spanning 1 to 5000 µg/kg SEM. The R² value of the standard curve was 0.9994 (Figure 2D).

### 3.4. Comparison of QuEChERS-HILIC-MS/MS with Conventional Method

Accuracy assessment of the proposed method involved comparison with the conventional method established by Li et al. with slight modification [26] (detailed information about the conventional detection method is listed in the Supplementary Materials). In the conventional method, the use of 2-nitrobenzaldehyde as a derivatization reagent enhances SEM retention on reversed-phase chromatography columns. This improvement minimizes interference from low-quality charge ratio substances in the matrix, providing a slight advantage. Under the conditions of this study, the LOD and LOQ for the conventional method were 0.3 μg/kg and 0.8 μg/kg, respectively. In contrast, the LOD and LOQ for the QuEChERS-HILIC-MS/MS method were 1.0 μg/kg and 3.0 μg/kg, respectively. The established method demonstrated a comparable sensitivity to RPLC-MS/MS (Table 1), making it adequate for routine SEM detection and analysis.

To assess the advancements of the QuEChERS-HILIC-MS/MS method, reagent usage, the analysis procedure, and the total workflow time were compared with the conventional method. The conventional method relied on organic solvents such as dimethyl sulfoxide, methanol, and ethyl acetate, while the proposed method used only acetonitrile in smaller quantities, aligning with eco-friendly requirements. Additionally, the conventional method required 16 h for derivatization, resulting in a total workflow time of approximately 18 h. In contrast, the QuEChERS-HILIC-MS/MS method eliminated the need for derivatization, reducing the total workflow time to about 3 h through two steps of extraction and dSPE purification. The common method also required the preparation of reagents such as hydrochloric acid, dipotassium hydrogen phosphate, and 2-nitrofurfural solution, which had to be freshly prepared. The QuEChERS-HILIC-MS/MS method allowed for pre-weighing of the necessary additives, which could be directly incorporated into the workflow, simplifying the process and enabling batch processing. A detailed comparison of the two methods is provided in Table 2.

### 3.5. Detection of SEM in Crustaceans by QuEChERS-HILIC-MS/MS

**Figure 3:**
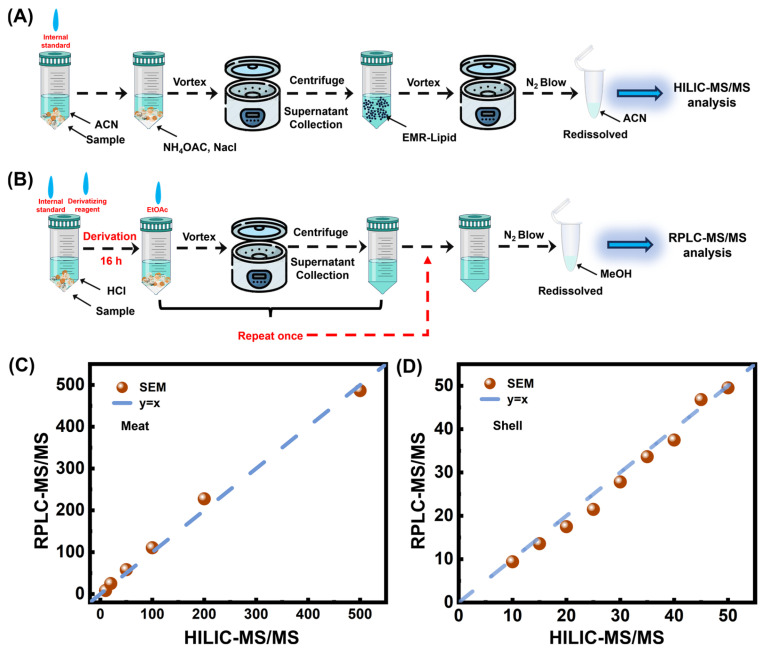
Detection of SEM in *Macrobrachium rosenbergii*. (**A**) Workflow for SEM analysis in *M. rosenbergii* using the established method. (**B**) Workflow using the conventional method. (**C**,**D**) Comparison of SEM content in meat (**C**) and shell (**D**) between the established and conventional detection methods.

*Macrobrachium rosenbergii*, a commonly farmed crustacean, is frequently subjected to illegal NFZ treatment. Thus, this species was chosen to validate the accuracy of the QuEChERS-HILIC-MS/MS method. Equal amounts of SEM were added to both blank meat and shell samples from *M. rosenbergii*. The workflows for the established method and the conventional method are illustrated in Figure 3A,B. The correlation between the two methods for SEM content measurement was analyzed. The results showed that the error for shrimp meat was less than 8%, while the error for shrimp shells was less than 10% (Figure 3C,D), demonstrating the accuracy of our proposed method.

**Figure 4:**
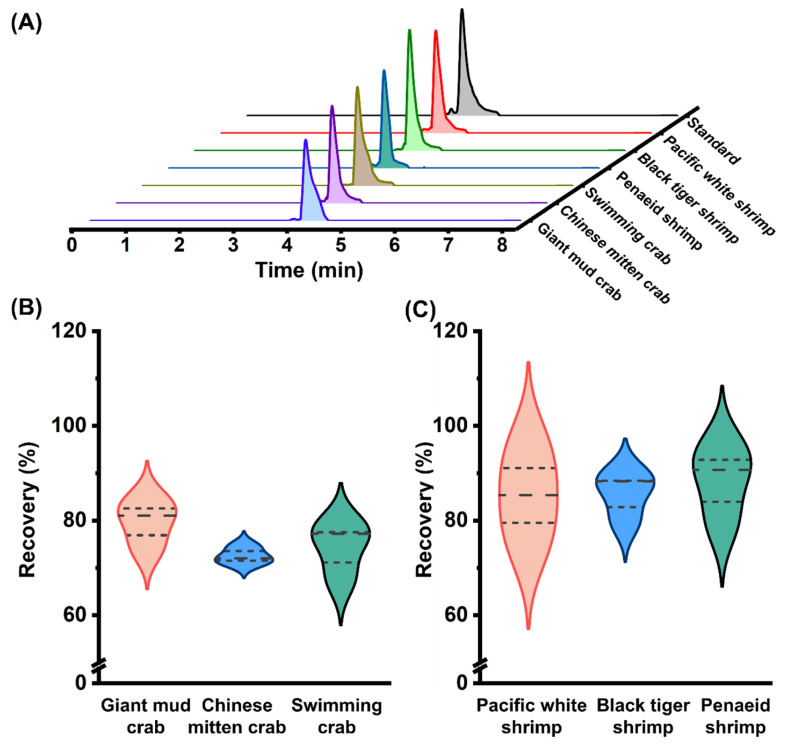
Detection of SEM in various crustacean species. (**A**) Extracted ion chromatograms of SEM in various shrimp and crab species. (**B**,**C**) Recovery for shrimp (**B**) and crab (**C**) spiked with fixed SEM concentrations.

Additionally, a study was conducted to examine the universality of the QuEChERS-HILIC-MS/MS method. Various crustacean species, including shrimp (*Litopenaeus vannamei*, *Metapenaeus ensis*, and *Penaeus monodon*) and crabs (*Portunus trituberculatus*, *Eriocheir sinensis*, and *Scylla serrata*), were selected for further research. A fixed SEM concentration was added to matrix extracts from these species, followed by QuEChERS-HILIC-MS/MS analysis. The chromatographic peaks for SEM were clearly detected across different shrimp and crab species (Figure 4A). Also, the recovery for the different crustacean samples were measured, ranging from 71.1% to 95.3% (Figure 4B,C). These results confirmed the universality of the QuEChERS-HILIC-MS/MS method and further validated its accuracy and reliability.

### 3.6. Metabolism of SEM in Macrobrachium rosenbergii Treated with NFZ

**Figure 5:**
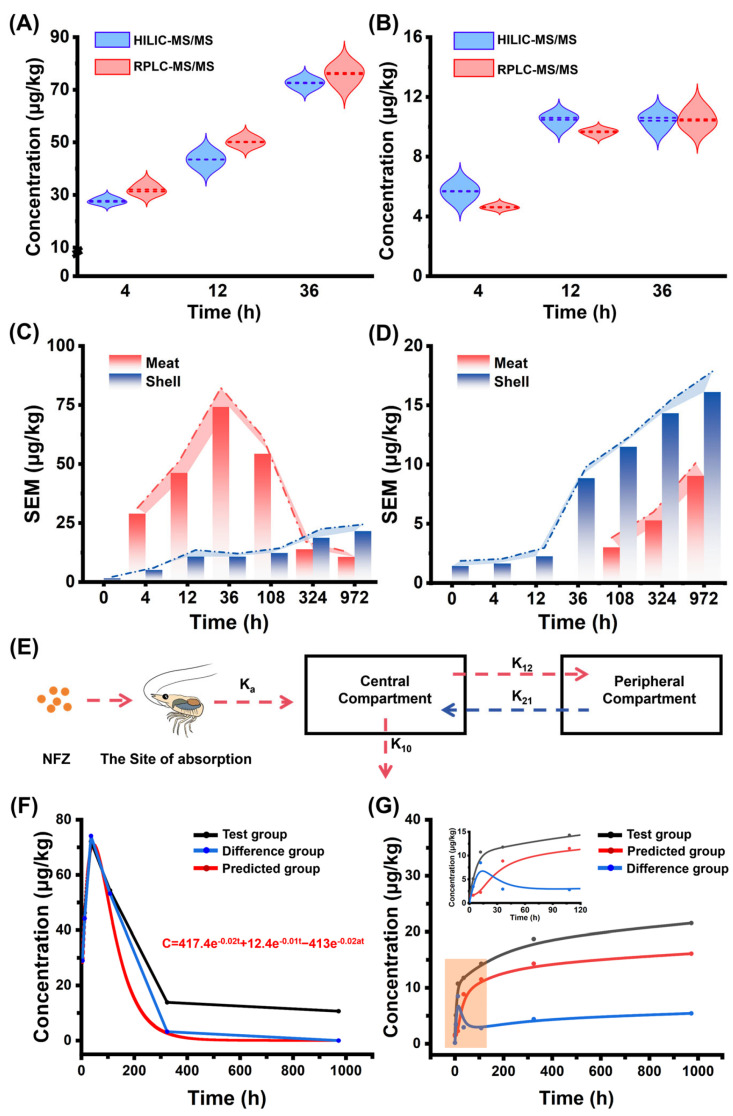
Metabolism of SEM in *Macrobrachium rosenbergii* treated with NFZ. (**A**,**B**) SEM content comparison between NFZ-treated (**A**) and control groups (**B**) using the established and conventional methods at 4, 12, and 36 h. (**C**,**D**) Changes in SEM content in NFZ-treated (**C**) versus control groups (**D**) at different time points. (**E**) Kinetic model for SEM metabolism. (**F**,**G**) Simulated kinetic curves for SEM metabolism generated by NFZ (**F**) and changes due to endogenous presence (**G**).

The proposed QuEChERS-HILIC-MS/MS method was applied to investigate changes in SEM content in the meat and shells of *Macrobrachium rosenbergii* following an NFZ drug bath. Samples collected after 4, 12, and 36 h of exposure were analyzed using both the proposed and conventional methods (Figure 5A,B). The results further validated the accuracy of the proposed method. The SEM metabolism was assessed, showing that NFZ is rapidly metabolized in *M. rosenbergii* (Figure 5C,D). A significant peak in the SEM response was detected 4 h after exposure, increasing over time and reaching a maximum concentration of 74.1 μg/kg in shrimp meat after 36 h. The SEM content in the shells also increased, rising from 1.6 μg/kg initially to 21.5 μg/kg. In contrast, the control group displayed a gradual increase in SEM content in shrimp meat, detectable at 108 h and peaking at 9.04 μg/kg at 972 h. The SEM was consistently detected in shrimp shells throughout the study period, with a steady increase over time.

Furthermore, a non-invasive two-compartment kinetic model was employed to study this process (Figure 5E). Data fitting with the experimental results from the medicated bath group revealed first-order kinetic parameters. Observed and predicted values were compared (Figure 5F), showing strong agreement within the first 108 h. However, deviations emerged as time progressed. To address this, SEM content from the control group was subtracted from the test group to account for endogenous SEM interference. The results aligned closely with the model predictions (AIC = −29.562), confirming the two-compartment model as appropriate for quantifying SEM accumulation in *M. rosenbergii*. Comprehensive data regarding the pharmacokinetic parameters are presented in Tables S3–S5, indicating that the half-life alpha (t1/2*α*) and half-life beta (t1/2*β*) are 42.8 h and 69.3 h, respectively. Notably, there are slight variations in the drug’s half-life as concluded by previous reports [27,28]. These variations may be attributed to differences in the experimental subjects as well as the methods of administration employed. An unexpected upward trend in SEM content within shrimp shells, diverging from the anticipated drug metabolism model, was investigated. The relationship between SEM levels in the test group and control group and the difference between the two were examined (Figure 5G). After 108 h, SEM levels in all groups gradually increased. Unlike the treatment group (black line) and control group (red line), the difference in SEM content (blue line) fluctuated, initially declining, then increasing, and declining again between 12 and 972 h.

It was hypothesized that the medicated bath triggered a specific mechanism of SEM absorption or formation in the shells before 12 h, leading to a rapid increase in the treatment group compared to the control [28]. As concentrations in the control group rose and the rate of increase in the treatment group changed, the disparity narrowed. After 36 h, the treatment group’s concentration rose rapidly again, suggesting a second wave of absorption, with SEM transferring from the meat to the shell. Between 324 and 972 h, a downward trend emerged, indicating that endogenous factors influenced the SEM content in the later stages. This analysis provides valuable insights into the complex dynamics of SEM accumulation in shrimp shells under NFZ-medicated bath conditions.

## 4. Conclusions

In summary, a novel method for SEM detection in crustaceans was developed using modified QuEChERS coupled with HILIC-MS/MS analysis. The established method demonstrated excellent linearity and accuracy. Compared to the conventional detection method, the proposed approach is eco-friendly and significantly reduces analysis time. The application of this method to detect SEM in *Macrobrachium rosenbergii* yielded results consistent with those obtained using the conventional method. Additionally, the established method was validated for universal application in detecting SEM across various crustacean species. Furthermore, the method was successfully employed to study SEM metabolism in an NFZ drug bath. Overall, the developed method provides a reliable and accurate approach for seafood quality and safety testing and offers valuable insights for pharmacokinetic studies of banned substances.

## Figures and Tables

**Table 1:** Calibration equations, limits of detection (LODs), and limits of quantitation (LOQs) for the established QuEChERS-HILIC-MS/MS method and the conventional RPLC-MS/MS.

	Calibration Curve	LOD	LOQ
RPLC-MS/MS	y = 1.1011x − 0.0087	0.3 μg/kg	0.8 μg/kg
HILIC-MS/MS	y = 1.0223x − 0.1512	1.0 μg/kg	3.0 μg/kg

**Table 2:** Comparison of the established QuEChERS-HILIC-MS/MS method and the conventional method, including the reagents used, analysis procedure, and workflow time.

	Chemical Reagents	Procedure	Time Consumption
RPLC-MS/MS	Nitrobenzaldehyde, Methanol, Lithium dihydrogen phosphate, Ethyl acetate, N-hexane, Hydrochloric acid, Dimethyl sulfoxide (DMSO)	Liquid extraction, Derivatization, Purification, Enrichment	18 h
HILIC-MS/MS	Acetonitrile, Formic acid	Extraction, Purification, Enrichment	3 h

## Data Availability

The original contributions presented in this study are included in this article/Supplementary Materials. Further inquiries can be directed to the corresponding author.

## Supplementary Materials

The following supporting information can be downloaded at https://www.mdpi.com/article/10.3390/foods14030541/s1: Figure S1: The impact of acetonitrile addition on SEM recovery in shrimp shell; Figure S2: Comparison of intensity in the SEM analysis of the standard solution and the sample extract on a C18 column; Table S1: Optimized MRM transitions, cone voltages, and collision energies for analysis of SEM by HILIC-MS/MS; Table S2: Extraction efficiency, matrix effect, recovery, and intra- and inter-day precision for the determination of SEM in *Macrobrachium rosenbergii* samples; Table S3: Primary kinetic parameters of non-intravenous two-compartment model; Table S4: Detailed kinetic parameters of a two-compartment model; Table S5: Pharmacokinetic parameters of a two-compartment model.

## Abbreviations

The following abbreviations are used in this manuscript:

SEM	Semicarbazide
NFZ	Nitrofurazone
RPLC-MS/MS	Reversed-phase liquid chromatography–tandem mass spectrometry
HILIC-MS/MS	Hydrophilic interaction liquid chromatography–tandem mass spectrometry
QuEChERS	Quick, Easy, Cheap, Effective, Rugged, and Safe
