# Supplementary material for: A Novel Strategy for the Detection of Semicarbazide in Crustaceans by Modified QuEChERS Coupled with Hydrophilic Interaction Liquid Chromatography–Tandem Mass Spectrometry
Source: Foods. 2025 Feb 6;14(3):541. doi: 10.3390/foods14030541 (PMC11816364; doi:10.3390/foods14030541)
Supplement: Supplementary file 1 [file foods-14-00541-s001.zip › supporting information.docx]

**A Novel Strategy for Detection of Semicarbazide in Crustaceans by Modified QuEChERS Coupled with Hydrophilic Interaction Liquid Chromatography -Tandem Mass Spectrometry**

**Siyuan Wu^a,b^, Yang Feng^b,c,*^, Shengjun Chen^a,b,c,*^, Yongqiang Zhao^b,c^, Chunsheng Li^b,c^, Jianchao Deng^b,c^, Di Wang^b,c^**

^a^College of Food Science, Shanghai Ocean University, Shanghai 201306, China

^b^Key Laboratory of Aquatic product, Ministry of Agriculture and Rural affairs, National R&D Center for Aquatic Product Processing, South China Sea Fisheries Research Institute, Chinese Academy of Fishery Sciences, Guangzhou 510330, China

^c^Key Laboratory of Efficient Utilization and Processing of Marine Fishery Resources of Hainan Province, Sanya Tropical Fisheries Research Institute, Sanya 572426, China

Table of Contents

| Page S3-5 | Materials and methods: crustacean samples acquisition, culture method of *Macrobrachium rosenbergii*, and conventional method for SEM analysis. |
| --- | --- |
| Page S6 | Figure S1. The impact of acetonitrile addition on SEM recovery in shrimp shells. |
| Page S7 | Figure S2. Comparison of intensity in the SEM analysis of standard solution (A) and sample extract (B) on a C18 column. |
| Page S8 | Table S1. Optimized MRM transitions, cone voltages, and collision energies for analysis of SEM by HILIC-MS/MS. |
| Page S9 | Table S2. Extraction efficiency, matrix effect, recovery, intra- and inter-day precision for the determination of SEM in *Macrobrachium rosenbergii* samples. |
| Page S10 | Table S3. Primary kinetic parameters of non-intravenous two-compartment model. |
| Page S11 | Table S4. Detailed kinetic parameters of a two-compartment model. |
| Page S12 | Table S5. Pharmacokinetic parameters of a two-compartment model. |
| Page S13 | References. |

**Material and method**

**Crustacean samples acquisition**

Giant river prawn (*Macrobrachium rosenbergii*), pacific white shrimp (*Litopenaeus vannamei*), penaeid shrimp (*Metapenaeus ensis*), black tiger shrimp (*Penaeus monodon*), swimming crab (*Portunus trituberculatus*), Chinese mitten crab (*Eriocheir sinensis*), and giant mud crab (*Scylla serrata*) were purchased from a local seafood supplier in the Haizhu District of Guangzhou City, China. All crustacean samples acquired were healthy adult individuals that were not pregnant. The weights of the various crustacean species are as follows: giant river prawn approximately 15 g, pacific white shrimp about 20 g, black tiger shrimp around 24 g, swimming crab about 200 g, sword prawn approximately 250 g, and Chinese mitten crab around 100 g. While there is significant variation in body size among different species, individuals of the same species were carefully selected to be of similar size.

**Culture method of *Macrobrachium rosenbergii***

The cultured *Macrobrachium rosenbergii* were sourced from an aquaculture facility in Guangzhou. No nitrofurazone (NFZ) drugs were administered during the growth period, and there is no history of NFZ abuse associated with this aquaculture facility. Mature individuals were selected based on the completeness of their antennae and legs, as well as their lively and active behavior, to assess their maturity. After purchase, the shrimps were continuously supplied with oxygen and transported to the laboratory in a vehicle. They were then temporarily housed in an indoor recirculating water tank. Prior to the commencement of the experiment, the shrimp were fasted for two days, and any deceased individuals were promptly removed.

The culture of *Macrobrachium rosenbergii* is maintained at a room temperature of 20°C, with continuous aeration provided by an oxygen pump to sustain a maximum dissolved oxygen level of 7.8 mg/L. The culture tank is equipped with disinfected plastic dividers to prevent aggression among the shrimp. Daily, 5 g of NFZ-free feed is added to the tank. This feed consists of 100 g of corn, 50 g of soybean, 40 g of fish meal, and trace amounts of mineral salts, maintaining a ratio of corn to soybean to fish meal of 10:5:4. Throughout the culture period, feces and uneaten food are removed using a siphon method, and any deceased shrimp are promptly discarded. Both the water and feed are analyzed using SEM before and after the official commencement of the experiment. On average, individuals with a mass of 12.0 g ± 2.0 g and a body length of 6 cm ± 1 cm are selected and placed into the culture tanks, where they are randomly grouped at a stocking density of approximately 60 individuals per cubic meter.

**Conventional method for SEM analysis**

The determination of SEM in shrimp follows the protocols established by Li *et al* with slight modification [1]. For shrimp meat, 2 g of the sample was accurately weighed and placed into a 50 mL centrifuge tube. Next, we add 0.05 mL of a working solution of the internal standard ^13^C-^15^N_2_-SEM at a concentration of 10 ng/mL and vortex mix for 50 s. Subsequently, we introduced 5 mL of hydrochloric acid solution and 0.15 mL of 2-nitrobenzaldehyde solution. After vortex mixing for an additional 50 s, we incubated the mixture in a constant-temperature water bath at 37°C in the dark for 16 h.

For sample extraction, we added dipotassium hydrogen phosphate (1 mM aqueous solution) to adjust the pH of the mixture to 7.0-7.5. Next, 4 mL of ethyl acetate was added and mixed thoroughly. The mixture was then centrifuged at 6000 rpm for 10 min, and the supernatant was collected. To ensure complete extraction, we repeated the aforementioned procedure and combined the supernatants. The combined solution was subjected to nitrogen blowdown at 40°C. We re-dissolved the residue in a 2 mmol/L ammonium acetate solution containing 0.1% formic acid and filtered it through a 0.45 μm filter membrane for analysis. The ACQUITY™ UPLC BEH C18 column (2.1 mm × 100 mm, 1.7 μm) was used for compound separation. The injection volume was 20 μL, and the sample chamber temperature was maintained at 4°C. The flow rate was set to 0.2 mL/min, with mobile phase A consisting of a 2 mmol/L ammonium acetate and mobile phase B being methanol. For MS analysis, the ionization mode was positive ion electrospray (ESI^+^), with a spray voltage of 3800 V, an auxiliary gas flow rate of 3 L/min, and an ion transfer capillary temperature of 350°C. The scanning mode employed was multiple reaction monitoring (MRM), utilizing the ion with the highest abundance for quantification (SEM, 209.0 > 166.0; ^13^C-^15^N_2_-SEM, 212.0 > 168.0). Qualitative analysis was conducted using characteristic ion fragments (SEM, 209.0 > 192.0; ^13^C-^15^N_2_-SEM, 212.0 > 195.0).

**Fig. S1. The impact of acetonitrile addition on SEM recovery in shrimp shells.
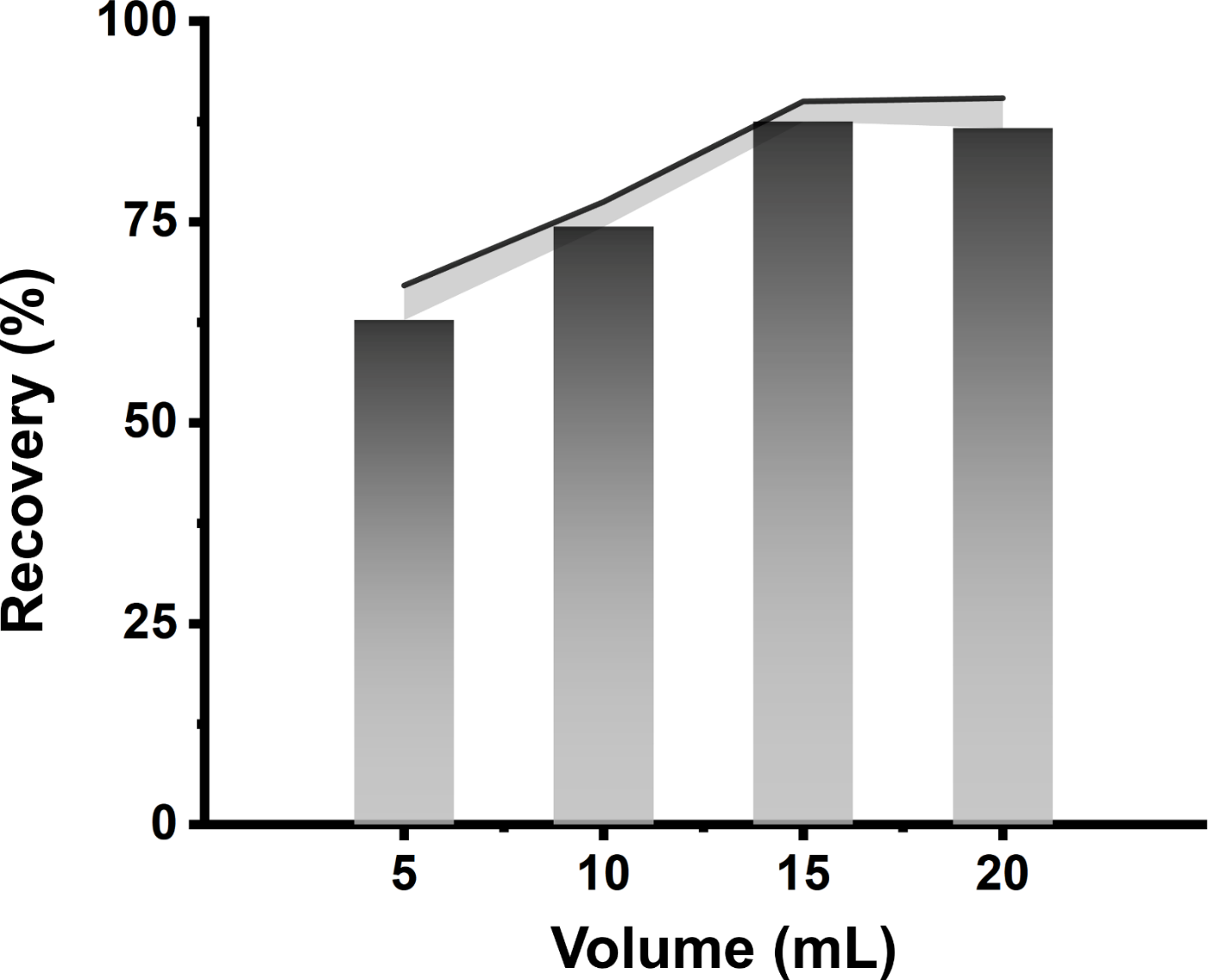
**

**Fig. S2. Comparison of intensity in the SEM analysis of standard solution (A) and sample extract (B) on a C18 column.
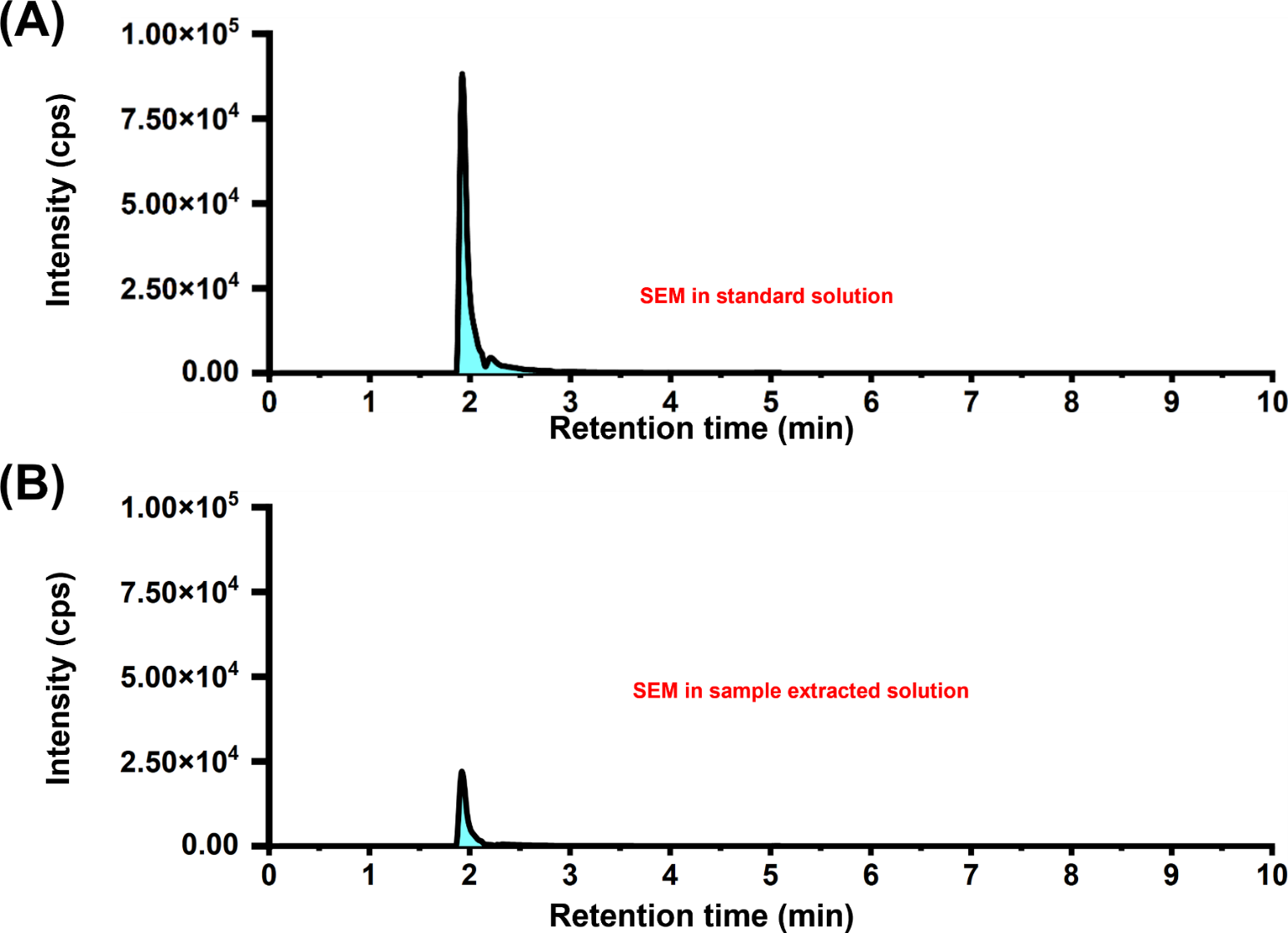
**

**Table. S1. Optimized MRM transitions, cone voltages, and collision energies for analysis of SEM by HILIC-MS/MS.**

| **Analyte** | **Parent ion (m/z)** | **Daught ion (m/z) *** | **Daught ion (m/z)** | **Q1** | **Q2** |
| --- | --- | --- | --- | --- | --- |
| SEM | 76.1 | 30.8 | 58.8 | 12 | 12 |
| ^13^C-^15^N_2_-SEM | 79.0 | 33.0 | 61.8 | 10 | 12 |

* Indicates that the daughter ion for quantitative analysis. Daughter ion without * was used for qualitative analysis.

**Table. S2. Extraction efficiency, matrix effect, recovery, intra- and inter-day precision for the determination of SEM in *Macrobrachium rosenbergii* samples.**

|  | | **Extraction efficiency (%)** | **Matrix effect (%)** | **Recovery (%)** | **Inter-day (RSD, %, n=5)** | **Intra-day (RSD, %, n=5)** |
| --- | --- | --- | --- | --- | --- | --- |
| HILIC-MS/MS | Low | 78.2±4.6 | 75.7±5.9 | 76.2±4.1 | 2.9±1.5 | 9.2±2.8 |
|  | Medium | 87.6±5.7 | 82.3±4.1 | 85.4±6.8 | 5.6±1.2 | 6.7±2.2 |
|  | High | 95.4±8.5 | 92.1±3.4 | 96.7±5.0 | 6.2±3.1 | 7.3±3.7 |
| RPLC-MS/MS | Low | - | - | 78.8±5.4 | 2.3±2.4 | 5.0±2.5 |
|  | Medium | - | - | 94.3±4.0 | 5.0±0.8 | 3.8±2.0 |
|  | High | - | - | 105.2±3.1 | 5.8±2.0 | 4.8±4.5 |

**Table. S3. Primary kinetic parameters of non-intravenous two-compartment model.**

| **Primary Parameter** | **Parameter Value** | **Fitting Result** | **Value** |
| --- | --- | --- | --- |
| A | 417.352 | AIC | -29.562 |
| *α* | 0.016 | AE | 3.363 |
| B | 12.425 | RE | 0.001 |
| *β* | 0.01 | R^2 | 1 |
| K | 412.985 | SBC | -30.812 |
| Ka | 0.024 | Fitting times | 464 |

**Table. S4. Detailed kinetic parameters of a two-compartment model.**

| **Parameter** | **Unit** | **Parameter Value** | **Description** |
| --- | --- | --- | --- |
| Ka | 1/h | 0.024 | Absorption rate constant: The rate constant of drug absorption. |
| K12 | 1/h | 0.01 | Rate constant from compartment 1 to compartment 2: The rate constant of transfer from the central compartment to the peripheral compartment. |
| K21 | 1/h | 0.01 | Rate constant from compartment 2 to compartment 1: The rate constant of transfer from the peripheral compartment back to the central compartment. |
| K10 | 1/h | 0.006 | Rate constant from compartment 1 to compartment 0: The elimination rate constant from the central compartment. |
| Tlag | h | 0 | Lag time: The time delay between administration and the start of absorption. |
| t1/2Ka | h | 28.614 | Half-life of absorption: The time it takes for the drug absorption process to decrease by half. |
| Tmax | h | 36 | Time to reach Maximum concentration: The time it takes to reach the peak concentration. |
| t1/2*α* | h | 42.801 | Half-life alpha: The time it takes for the drug concentration in the central compartment to decrease by half. |
| t1/2*β* | h | 69.315 | Half-life beta: The time it takes for the drug concentration in the peripheral compartment to decrease by half. |

**Table. S5. Pharmacokinetic parameters of a two-compartment model.**

| **Parameter** | **Unit** | **Value** | **Description** |
| --- | --- | --- | --- |
| V1/F | L/kg | 0.14 | Volume of the central compartment per unit of dosage: Represents the volume of drug distribution in the central compartment (usually blood and highly perfused tissues). |
| CL/F | L/h/kg | 0.001 | Clearance per unit of dosage: Represents the amount of drug cleared from the body per unit time. |
| AUC(0-t) | mg/L*h | 21770 | Area Under the Curve from time 0 to t: Represents the total exposure of the drug from the start of administration to time t. |
| AUC(0-∞) | mg/L*h | 23620 | Area Under the Curve from time 0 to infinity: Represents the total exposure of the drug from the start of administration to an infinite time. |
| AUMC(0-t) | mg/L*h | 6275317 | Area Under the Moment Curve from time 0 to t: Used to calculate the mean residence time. |
| AUMC(0-∞) | mg/L*h | 8319331 | Area Under the Moment Curve from time 0 to infinity. |
| MRT(0-t) | h | 288.78 | Mean Residence Time from time 0 to t: Represents the average residence time of the drug from the start of administration to time t. |
| MRT(0-∞) | h | 381.95 | Mean Residence Time from time 0 to infinity: Represents the average residence time of the drug from the start of administration to an infinite time. |
| Zeta | 1/h | 0.006 | Zeta value: Characterizes the tail of the residence time distribution, used to describe the heterogeneity of drug distribution in the body. |
| t1/2z | h | 116.64 | Half-life of Zeta: The half-life of the Zeta value. |
| Vz/F | L/kg | 0.155 | Volume of the peripheral compartment per unit of dosage: Represents the volume of drug distribution in the peripheral compartment. |
| CLz/F | L/h/kg | 0.001 | Clearance of the peripheral compartment per unit of dosage: Represents the clearance rate of the drug from the peripheral compartment. |

**Reference**

1. Li, J.; Wang, D.; Chen, S.; Gao, F.; Li, C.; Feng, Y.; Deng, J. Integrated Metabolomic and Transcriptomic Analysis Reveal the Production Mechanism of Semicarbazide in Macrobrachium Rosenbergii Under Urea Conditions. *Foods* **2024**, *13*, doi:10.3390/foods13233817.
